# Supplementary material for: A comparison of psychological characteristics in people with knee osteoarthritis from Japan and Australia: A cross-sectional study
Source: PLoS One. 2022 May 5;17(5):e0267877. doi: 10.1371/journal.pone.0267877 (PMC9070885; doi:10.1371/journal.pone.0267877)
Supplement: S1 Table — (PDF) [file pone.0267877.s001.pdf]

S1 Table. The association of psychological outcomes with countries, age and BMI based on multiple regression analysis

		Unstandardized β (95% CI)	Standardized β	Adjusted R^2
**Depression**	**Unadjusted Model**			
	Japan vs Australia (reference)	-0.75 (-2.18, 0.69)	-0.07	0.00
	**Adjusted Model 1**			
	Japan vs Australia (reference)	-0.32 (-1.91, 1.28)	-0.03	0.00
	Age	0.05 (-0.03, 0.14)	0.09	
	**Adjusted Model 2**			
	Japan vs Australia (reference)	-1.62 (-3.14, -0.09)**	-0.15	0.03
	BMI	0.15 (0.05, 0.24)*	0.21	
	**Adjusted Model 3**			
	Japan vs Australia (reference)	-1.05 (-2.68, 0.57)	-0.10	0.04
	Age	0.09 (0.00, 0.18)	0.15	
	BMI	0.17 (0.07, 0.27)**	0.24	
**Fear of movement**	**Unadjusted Model**			
	Japan vs Australia (reference)	0.77 (-0.22, 1.77)	0.10	0.04
	**Adjusted Model 1**			
	Japan vs Australia (reference)	0.48 (-0.62, 1.59)	0.06	0.01
	Age	-0.04 (-0.10, 0.02)	-0.09	
	**Adjusted Model 2**			
	Japan vs Australia (reference)	0.35 (-0.72, 1.41)	0.05	0.02
	BMI	0.07 (0.00, 0.14)*	0.15	
	**Adjusted Model 3**			
	Japan vs Australia (reference)	0.20 (-0.94, 1.34)	0.03	0.02
	Age	-0.23 (-0.09, 0.04)	-0.06	
	BMI	0.07 (-0.01, 0.14)	0.13	
**PCS total score**	**Unadjusted Model**			
	Japan vs Australia (reference)	-5.85 (-8.92, -2.79)**	-0.26	0.06
	**Adjusted Model 1**			
	Japan vs Australia (reference)	-6.70 (-10.11, -3.30)**	-0.30	0.07
	Age	-0.12 (-0.31, 0.08)	-0.09	
	**Adjusted Model 2**			

	Japan vs Australia (reference)	-7.18 (--10.52, -3.85)**	-0.32	0.08
	BMI	0.23 (-0.01, 0.46)	0.15	
	**Adjusted Model 3**			
	Japan vs Australia (reference)	-7.62 (-11.17, -4.08)**	-0.34	0.08
	Age	-0.08 (-0.28, 0.13)	-0.06	
	BMI	0.21 (-0.03, 0.45)	0.13	
**PCS Rumination**	**Unadjusted Model**			
	Japan vs Australia (reference)	-3.00 (-4.17, -1.83)**	-0.34	0.11
	**Adjusted Model 1**			
	Japan vs Australia (reference)	-3.02 (-4.32, -1.72)**	-0.35	0.11
	Age	-0.00 (-0.08, 0.07)	-0.01	
	**Adjusted Model 2**			
	Japan vs Australia (reference)	-3.22 (-4.50, -1.93)**	-0.37	0.11
	BMI	0.04 (-0.05, 0.13)	0.06	
	**Adjusted Model 3**			
	Japan vs Australia (reference)	-3.19 (-4.56, -1.83)**	-0.37	0.11
	Age	0.00 (-0.07, 0.08)	0.01	
	BMI	0.04 (-0.05, 0.13)	0.06	
**PCS Magnification**	**Unadjusted Model**			
	Japan vs Australia (reference)	-0.63 (-1.43, 0.16)	-0.11	0.01
	**Adjusted Model 1**			
	Japan vs Australia (reference)	-1.09 (-1.96, -0.22)*	-0.20	0.03
	Age	-0.06 (-0.11, -0.10)*	-0.19	
	**Adjusted Model 2**			
	Japan vs Australia (reference)	-1.04 (-1.90, -0.18)*	-0.19	0.03
	BMI	0.07 (0.01, 0.13)*	0.18	
	**Adjusted Model 3**			
	Japan vs Australia (reference)	-1.34 (-2.24, -0.43)**	-0.24	0.04
	Age	-0.05 (-0.10, 0.00)	-0.16	
	BMI	0.06 (-0.00, 1.12)	0.15	
**PCS Helplessness**	**Unadjusted Model**			
	Japan vs Australia	-2.23 (-3.60, -0.85)**	-0.23	0.05

	(reference)			
	**Adjusted Model 1**			
	Japan vs Australia (reference)	-2.60 (-4.12, -1.08)**	-0.26	0.05
	Age	-0.05 (-0.14, 0.04)	-0.09	
	**Adjusted Model 2**			
	Japan vs Australia (reference)	-2.92 (-4.41, -1.44)**	-0.30	0.07
	BMI	0.12 (0.02, 0.22)*	0.17	
	**Adjusted Model 3**			
	Japan vs Australia (reference)	-3.09 (-4.67, -1.52)**	-0.32	0.06
	Age	-0.03 (-0.12, 0.06)	-0.05	
	BMI	0.11 (0.01, 0.22)*	0.16	

BMI; Body Mass Index, PCS; Pain catastrophizing scale, **p<0.01, *p<0.05
