# Supplementary material for: A comparison of psychological characteristics in people with knee osteoarthritis from Japan and Australia: A cross-sectional study
Source: PLoS One. 2022 May 5;17(5):e0267877. doi: 10.1371/journal.pone.0267877 (PMC9070885; doi:10.1371/journal.pone.0267877)
Supplement: Attachment — Submitted filename: Plos One 2022.docx [file pone.0267877.s003.docx]

This is interesting study comparing psychological aspect of people suffering with Osteoarthritis from 2 developed countries; Australia and Japan.

My only concern is the current data analysis is not sufficient to draw the conclusion as suggested. I would suggest the authors to run linear regression to look at whether the higher Pain catastrophizing rumination reported by Japanese as compared to Australia is influence/confound by other factors such age and BMI.

Example of Table is as below:

Multiple Linear Regression Model

|  | β, Mean Differences, (95% CI) | | | | | |
| --- | --- | --- | --- | --- | --- | --- |
|  | Depression | Fear of movement | Pain catastrophizing | Rumination | Magnification | Helplessness |
| **Unadjusted Model** |  |  |  |  |  |  |
| *Japan vs Australia (reference)* |  |  |  |  |  |  |
| **Adjusted Model 1** |  |  |  |  |  |  |
| *Japan vs Australia (reference)* |  |  |  |  |  |  |
| *Age* |  |  |  |  |  |  |
| **Adjusted Model 2** |  |  |  |  |  |  |
| *Japan vs Australia (reference)* |  |  |  |  |  |  |
| *BMI* |  |  |  |  |  |  |
| **Adjusted Model 3** |  |  |  |  |  |  |
| *Japan vs Australia (reference)* |  |  |  |  |  |  |
| *Age* |  |  |  |  |  |  |
| *BMI* |  |  |  |  |  |  |
