# Supplementary material for: A comparison of psychological characteristics in people with knee osteoarthritis from Japan and Australia: A cross-sectional study
Source: PLoS One. 2022 May 5;17(5):e0267877. doi: 10.1371/journal.pone.0267877 (PMC9070885; doi:10.1371/journal.pone.0267877)
Supplement: Attachment — Submitted filename: Response to reviewers.docx [file pone.0267877.s004.docx]

Response to reviewers

My only concern is the current data analysis is not sufficient to draw the conclusion as suggested. I would suggest the authors to run linear regression to look at whether the higher Pain catastrophizing rumination reported by Japanese as compared to Australia is influence/confound by other factors such age and BMI.

Answer

We performed multiple regression analysis as suggested (S1 Table). In most models, age and BMI had no significant associations with psychological outcomes, or even if there were significant associations, they were extremely small. These results do not show any inconsistency with the results shown in Table 2.

We added the following sentences from line 166 to 170 and S1 Table.

“We performed an additional analysis to check the contribution of age and BMI as confounders in the association between country and psychological outcomes using multiple regression analysis (S1 Table). Age and/or BMI made either no contribution to the models or their contribution was to slightly strengthen the association between country and psychological outcomes.”

S1 Table. The association of psychological outcomes with countries, age and BMI based on multiple regression analysis

|  |  | Unstandardized β (95% CI) | Standardized β | Adjusted R^2^ |
| --- | --- | --- | --- | --- |
| **Depression** | **Unadjusted Model** |  |  |  |
|  | *Japan vs Australia*  *(reference)* | -0.75 (-2.18, 0.69) | -0.07 | 0.00 |
|  | **Adjusted Model 1** |  |  |  |
|  | *Japan vs Australia*  *(reference)* | -0.32 (-1.91, 1.28) | -0.03 | 0.00 |
|  | *Age* | 0.05 (-0.03, 0.14) | 0.09 |  |
|  | **Adjusted Model 2** |  |  |  |
|  | *Japan vs Australia*  *(reference)* | -1.62 (-3.14, -0.09)** | -0.15 | 0.03 |
|  | *BMI* | 0.15 (0.05, 0.24)* | 0.21 |  |
|  | **Adjusted Model 3** |  |  |  |
|  | *Japan vs Australia*  *(reference)* | -1.05 (-2.68, 0.57) | -0.10 | 0.04 |
|  | *Age* | 0.09 (0.00, 0.18) | 0.15 |  |
|  | *BMI* | 0.17 (0.07, 0.27)** | 0.24 |  |
| **Fear of movement** | **Unadjusted Model** |  |  |  |
|  | *Japan vs Australia*  *(reference)* | 0.77 (-0.22, 1.77) | 0.10 | 0.04 |
|  | **Adjusted Model 1** |  |  |  |
|  | *Japan vs Australia*  *(reference)* | 0.48 (-0.62, 1.59) | 0.06 | 0.01 |
|  | *Age* | -0.04 (-0.10, 0.02) | -0.09 |  |
|  | **Adjusted Model 2** |  |  |  |
|  | *Japan vs Australia*  *(reference)* | 0.35 (-0.72, 1.41) | 0.05 | 0.02 |
|  | *BMI* | 0.07 (0.00, 0.14)* | 0.15 |  |
|  | **Adjusted Model 3** |  |  |  |
|  | *Japan vs Australia*  *(reference)* | 0.20 (-0.94, 1.34) | 0.03 | 0.02 |
|  | *Age* | -0.23 (-0.09, 0.04) | -0.06 |  |
|  | *BMI* | 0.07 (-0.01, 0.14) | 0.13 |  |
| **PCS** | **Unadjusted Model** |  |  |  |
|  | *Japan vs Australia*  *(reference)* | -5.85 (-8.92, -2.79)** | -0.26 | 0.06 |
|  | **Adjusted Model 1** |  |  |  |
|  | *Japan vs Australia*  *(reference)* | -6.70 (-10.11, -3.30)** | -0.30 | 0.07 |
|  | *Age* | -0.12 (-0.31, 0.08) | -0.09 |  |
|  | **Adjusted Model 2** |  |  |  |
|  | *Japan vs Australia*  *(reference)* | -7.18 (--10.52, -3.85)** | -0.32 | 0.08 |
|  | *BMI* | 0.23 (-0.01, 0.46) | 0.15 |  |
|  | **Adjusted Model 3** |  |  |  |
|  | *Japan vs Australia*  *(reference)* | -7.62 (-11.17, -4.08)** | -0.34 | 0.08 |
|  | *Age* | -0.08 (-0.28, 0.13) | -0.06 |  |
|  | *BMI* | 0.21 (-0.03, 0.45) | 0.13 |  |
| **Rumination** | **Unadjusted Model** |  |  |  |
|  | *Japan vs Australia*  *(reference)* | -3.00 (-4.17, -1.83)** | -0.34 | 0.11 |
|  | **Adjusted Model 1** |  |  |  |
|  | *Japan vs Australia*  *(reference)* | -3.02 (-4.32, -1.72)** | -0.35 | 0.11 |
|  | *Age* | -0.00 (-0.08, 0.07) | -0.01 |  |
|  | **Adjusted Model 2** |  |  |  |
|  | *Japan vs Australia*  *(reference)* | -3.22 (-4.50, -1.93)** | -0.37 | 0.11 |
|  | *BMI* | 0.04 (-0.05, 0.13) | 0.06 |  |
|  | **Adjusted Model 3** |  |  |  |
|  | *Japan vs Australia*  *(reference)* | -3.19 (-4.56, -1.83)** | -0.37 | 0.11 |
|  | *Age* | 0.00 (-0.07, 0.08) | 0.01 |  |
|  | *BMI* | 0.04 (-0.05, 0.13) | 0.06 |  |
| **Magnification** | **Unadjusted Model** |  |  |  |
|  | *Japan vs Australia*  *(reference)* | -0.63 (-1.43, 0.16) | -0.11 | 0.01 |
|  | **Adjusted Model 1** |  |  |  |
|  | *Japan vs Australia*  *(reference)* | -1.09 (-1.96, -0.22)* | -0.20 | 0.03 |
|  | *Age* | -0.06 (-0.11, -0.10)* | -0.19 |  |
|  | **Adjusted Model 2** |  |  |  |
|  | *Japan vs Australia*  *(reference)* | -1.04 (-1.90, -0.18)* | -0.19 | 0.03 |
|  | *BMI* | 0.07 (0.01, 0.13)* | 0.18 |  |
|  | **Adjusted Model 3** |  |  |  |
|  | *Japan vs Australia*  *(reference)* | -1.34 (-2.24, -0.43)** | -0.24 | 0.04 |
|  | *Age* | -0.05 (-0.10, 0.00) | -0.16 |  |
|  | *BMI* | 0.06 (-0.00, 1.12) | 0.15 |  |
| **Helplessness** | **Unadjusted Model** |  |  |  |
|  | *Japan vs Australia*  *(reference)* | -2.23 (-3.60, -0.85)** | -0.23 | 0.05 |
|  | **Adjusted Model 1** |  |  |  |
|  | *Japan vs Australia*  *(reference)* | -2.60 (-4.12, -1.08)** | -0.26 | 0.05 |
|  | *Age* | -0.05 (-0.14, 0.04) | -0.09 |  |
|  | **Adjusted Model 2** |  |  |  |
|  | *Japan vs Australia*  *(reference)* | -2.92 (-4.41, -1.44)** | -0.30 | 0.07 |
|  | *BMI* | 0.12 (0.02, 0.22)* | 0.17 |  |
|  | **Adjusted Model 3** |  |  |  |
|  | *Japan vs Australia*  *(reference)* | -3.09 (-4.67, -1.52)** | -0.32 | 0.06 |
|  | *Age* | -0.03 (-0.12, 0.06) | -0.05 |  |
|  | *BMI* | 0.11 (0.01, 0.22)* | 0.16 |  |

**p<0.01, *p<0.05
